# Lateral Flow Serodiagnosis in the Double-Antigen Sandwich Format: Theoretical Consideration and Confirmation of Advantages

**DOI:** 10.3390/s21010039

**Published:** 2020-12-23

**Authors:** Dmitriy V. Sotnikov, Anatoly V. Zherdev, Boris B. Dzantiev

**Affiliations:** A.N. Bach Institute of Biochemistry, Research Center of Biotechnology of the Russian Academy of Sciences, Leninsky Prospect 33, 119071 Moscow, Russia; zherdev@inbi.ras.ru (A.V.Z.); dzantiev@inbi.ras.ru (B.B.D.)

**Keywords:** point-of-care assay, membrane tests, immunochromatography, lateral flow immunoassay, immune response, detection of antibodies, antigen-antibody reactions, mathematical modelling, assay sensitivity, limit of detection

## Abstract

Determination of the presence in the blood of antibodies specific to the causative agent of a particular disease (serodiagnosis) is an effective approach in medical analytical chemistry. Serodiagnostics performed in the lateral flow immunoassay format (immunochromatography) meet the modern requirements for point-of-care testing and are supported by existing technologies of large-scale diagnostic tests production, thus increasing the amount of attention in a tense epidemiological situation. For traditional lateral flow serodiagnostics formats, a large number of nonspecific immunoglobulins in the sample significantly reduces the degree of detectable binding. To overcome these limitations, an assay based on the formation of immobilized antigen-specific antibody-labeled antigen complexes detection was proposed. However, the requirements for its implementation, providing maximum sensitivity, have not been established. This article describes the mathematical model for the above assay. The influence of the ratio of reagent concentrations on the analysis results is considered. It is noted that the formation of specific antibody complexes with several labeled antigens is the main limiting factor in reducing the detection limit, and methods are proposed to minimize this factor. Recommendations for the choice of the assay conditions, following from the analysis of the model, are confirmed experimentally.

## 1. Introduction

The presence of antibodies specific to the causative agent of a certain disease in the blood (serodiagnosis) indicates the contact between the organism and the pathogen, and, for many diseases, it is considered an effective diagnostic parameter [1,2,3]. The advantages of such diagnostics in comparison with the detection and identification of the pathogen itself consist in the relatively simple sampling, the reproducible results that do not depend on the choice of the sampling site or that slowly change for the patient over time, and the excluded need to grow the pathogen until it reaches the detectable concentration. Serodiagnostics is actively used for many widespread and socially significant infections, including tuberculosis, human immunodeficiency virus, and more [4,5,6,7,8]. Particular interest in this diagnostic method has arisen this year due to the coronavirus pandemic [9,10,11].

Serodiagnostics can be implemented in different formats; however, today two of them dominate in mass practice—microplate enzyme immunoassay and immunochromatographic analysis—which correspond to their leading place in relation to immunodiagnostics in general [12,13,14]. At the same time, recent years have seen the accelerated development of point-of-care diagnostic tools, which make it possible to carry out methodically simple testing directly at the sampling site and quickly obtain results [15,16,17,18]. This requirement is fully met by immunochromatographic analysis (lateral flow immunoassay). All reagents required for the assay are pre-applied to the test strip (a composite of several membranes). Contact of the sample with this test strip initiates the movement of liquid along the pores of the membranes and analytical reactions with the applied reagents, including reagents containing a colored label. Most often, nanodispersed gold particles are used as such a label due to their intense color, ease of preparation, and modification [19,20,21]. As a result of these processes, labeled colored immune complexes are formed in certain zones of the test strip. A visual assessment of the presence or absence of coloration of these zones allows us to make conclusions about the test results [22,23].

**Figure 1:**
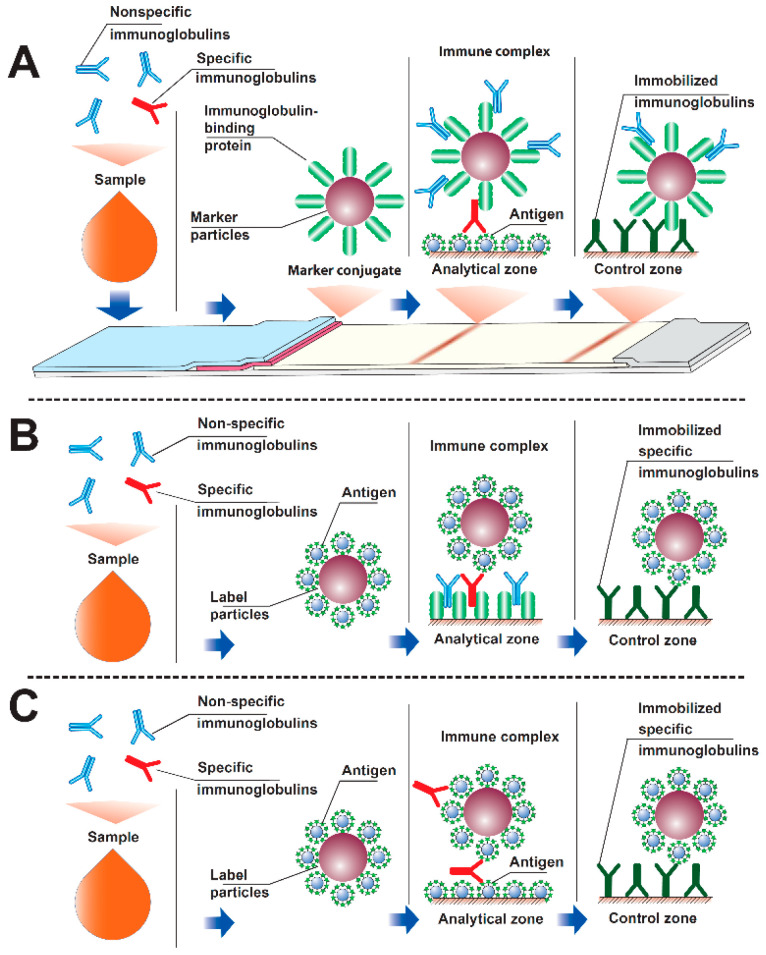
The three serodiagnostic immunochromatography formats: (**A**) with labeled immunoglobulin-binding protein and immobilized antigen in the analytical zone; (**B**) with labeled antigen and immobilized immunoglobulin-binding protein in the analytical zone; (**C**) with labeled antigen and immobilized antigen in the analytical zone (see additional comments in the paper).

For serodiagnostics, several formats are possible, differing in the location and labeling of immunoreagents. The most known format consists of immobilizing an antigen (specific for a given pathogen) in the analytical zone and labeling an immunoglobulin-binding reagent-anti-species antibodies, bacterial immunoglobulin-binding proteins (protein A, protein G), etc. [24,25,26,27,28,29]. The presence of specific antibodies to the pathogen in the sample leads to the formation of a detectable complex of immobilized antigen molecules, specific antibodies and labeled immunoglobulin-binding protein (Figure 1A). When carrying out such serodiagnostics, a reliable difference in results is achieved for samples containing and not containing specific antibodies. However, the intensity of the recorded color is often low, which prevents a correct assessment of the results obtained. The reason for this effect is that the labeled immunoglobulin-binding protein binds to all immunoglobulins in the tested blood or serum, of which only a few percent are specific to this pathogen [30,31]. The reaction of immunoglobulin-binding proteins with nonspecific immunoglobulins reduces the proportion of the label that is capable of participating in the formation of a detectable complex of antigen-specific antibody-immunoglobulin-binding protein label when the fluid passes through the analytical zone.

To overcome this limitation, an alternative immunochromatographic serodiagnostics format has been proposed, in which the label is conjugated to the antigen, and an immunoglobulin-binding protein is sorbed in the analytical zone [32,33,34,35,36,37]. This approach prevents the inclusion of the label in complexes with nonspecific immunoglobulins. The problem of signal attenuation, however, remains, because nonspecific immunoglobulins react with immunoglobulin-binding proteins in the analytical zone (Figure 1B). However, the sorption capacity of this zone on the test strip is significantly higher (about an order of magnitude) than the sorption capacity of the preparations of nanodispersed marker particles [36]. Therefore, the binding in the analytical zone of nonspecific antibodies to a lesser extent affects the formation of detectable complexes: immunoglobulin-binding protein–specific antibody-antigen-label.

To eliminate the influence of nonspecific immunoglobulins in the sample on the results of serodiagnostic immunochromatography, a third analysis format has been proposed, in which antigen is present both in the analytical zone and in combination with the label [33,38,39,40]. Due to several valencies of antibodies (2 for IgG, IgE and serum IgA, 10 for IgM), in this format, antigen-specific antibody-antigen-tag complexes can be formed in the analytical zone (Figure 1C). This scheme excludes the interaction of nonspecific immunoglobulins with components involved in the formation of a detectable complex. However, obstacles to the inclusion of a label in the detected complex exist in this case as well because a specific antibody can use its valences to bind only to labeled antigens. There are currently no recommendations in the literature to minimize these effects.

Taking into account the relevance of the requirement to minimize cases of false negative serodiagnostics results, the task of increasing its analytical sensitivity is becoming extremely important. However, the empirical variation of components concentrations and conditions of their interaction is an extremely laborious task. In addition, the choice for one test system may turn out to be far from optimal when switching to another test system (working with another antigenic preparation or with another infection). The process of developing test systems can be intensified using mathematical models because examining the model allows for identifying the key factors affecting the characteristics of the test system, understanding the reasons for the observed negative effects, and proposing ways to eliminate them. The practical application of the results of immunoanalytical systems modeling have been considered in a number of recent works and are summarized in previous reviews [41,42,43,44,45,46]; however, the regularities of the functioning of the test system with detected immobilized antigen-specific antibody-labeled antigen complexes have not been previously analyzed. Due to the above-mentioned potential advantages of such serodiagnostics, the presented work includes its theoretical consideration, identification, and verification of the regularities of the formation of detected complexes, and characteristics of factors influencing the analytical parameters of the test systems.

## 2. Materials and Methods

### 2.1. Reactants and Membranes

This study used recombinant protein A produced by Imtek (Moscow, Russia); Tween-20, sodium azide; N-(3-dimethylaminopropyl)-N’-ethylcarbodiimide (EDC), sulfo-N-hydroxysuccinimide (sulfo-NHS), and chloroauric from from Sigma-Aldrich (St. Louis, MO, USA); and bovine serum albumin (BSA) from Boval BioSolutions (Cleburne, TX, USA). Recombinant antigen of *Mycobacterium tuberculosis* with m.w. 38 kDa (Ag78, antigen 5, PhoS, Rv0934) came from Arista Biologicals Inc. (Allentown, PA, USA). Monoclonal antibodies HTM81 against the given protein came from the Center for Molecular Diagnostics and Therapy (Moscow, Russia). All salts were of chemical or analytical grade. Water was purified using a MilliQ unit from Millipore (Bedford, MA, USA).

To prepare immunochromatographic test systems, an mdi Easypack kit (Advanced Microdevices, Ambala Cantt, India) was used, which included working membranes CNPH90 with pores of 15 μm, a support for conjugate PT-R5, a membrane for applying samples FR1 (0.6), a final adsorbent membrane AP045, and a laminating protective film on an adhesive basis MT-1.

### 2.2. Gold Nanoparticles Preparation

To obtain gold nanoparticles with the expected average diameter near 20 nm, 2.95 mL of 0.34% chloroauric acid was added to 97.5 mL of boiling deionized water. The mixture was boiled for 2 min while stirring. Then 1.44 mL of 1% sodium citrate solution was added, stirred, boiled for 30 min, and cooled for storage at +4 °C.

### 2.3. Transmission Electron Microscopy

The gold nanoparticles preparation was applied onto nets (300 mesh) (Pelco International, Redding, CA, USA) covered with a polyvinyl formal film dissolved in chloroform. To obtain the images, a CX-100 microscope (Jeol, Tokyo, Japan) was used at an acceleration voltage of 80 kV. Photographs were digitally analyzed using the Image Tool software.

### 2.4. Immobilization of Rv0934 Antigen on Gold Nanoparticles

The antigen was dialyzed against 1000-fold of 10 mM carbonate buffer, pH = 9.0. To the gold nanoparticles solution (D_520_ = 1.0), 0.1 M K_2_CO_3_ was added until a pH = 9.0 was reached, and then the Rv0934 antigen at a concentration of 10 μg/mL was added. The mixture was incubated for 10 min at room temperature and stirred, after which a 10% aqueous BSA solution was added, for a final concentration of 0.25%.

The gold nanoparticles were separated from non-bound protein by 30-min centrifugation at 6.000× *g*, resuspending in 50 mM K-phosphate buffer, pH 7.4, with 0.1 M NaCl (PBS) containing 0.25% BSA. The preparations were stored at 4 °C.

### 2.5. Application of Reagents to Immunochromatographic Membranes

To form the analytical zone on the CNPH90 working membrane, the *M. tuberculosis* Rv0934 antigen was used. On 1 cm of the band, 1 μL of an antigen solution (1.0 mg/mL in 50 mM phosphate buffer, pH 7.4) was applied (according to Figure 1C “analytical zone” compartment). To form the control zone, antibodies HTM81 were applied to the working membrane at a concentration of 0.5 mg/mL (Figure 1C “control zone” compartment). The gold nanoparticles conjugate with the Rv0934 antigen (according to Figure 1C “Labeled antigen-nanoparticle” conjugate) was applied on the PT-R5 support at a dilution corresponding to D520 from 1.25 to 10.0, in a volume of 8 μL per cm of band. To apply the reagents, an IsoFlow dispenser (Imagene Technology, Hanover, NH, USA) was used.

### 2.6. Manufacturing of Immunochromatographic Test Systems

After applying the reagents, the membranes were dried in air at 20–22 °C for at least 20 h. A membrane composite was assembled, from which strips 3.5 mm wide were obtained, using an Index Cutter-1 guillotine cutter (A-Point Technologies, Gibbstown, NJ, USA). The test strips were placed in plastic cassettes, and the bags made of laminated aluminum foil were hermetically sealed with silica gel as a desiccant. Cutting and packing was implemented at 20–22 °C in a special room with a relative humidity of not more than 30%. The packaged test strips were stored at 20–22 °C.

### 2.7. Immunochromatographic Analysis 

The assay was carried out at room temperature. The bag was opened, and the test strip was placed on a horizontal surface. One drop of blood serum and then 3 drops of PBS containing 1% Tween-20 were added. After 10 min, the assay result was monitored. The binding of the label was quantitatively assessed using a portable digital video analyzer “Reflekom” (Synteco, Moscow, Russia).

### 2.8. Determination of Kinetic and Equilibrium Parameters of Interactions between Immunoreagents

To characterize the antigen-antibody interaction, a biosensor approach based on surface plasmon resonance was used. Measurements were performed on a BIAcore X (Biacore International, Uppsala, Sweden) using a CM5 chip with a carboxylated dextran-coated surface. Initially, the carboxyl groups of dextran were activated using a mixture of ethylcarbodiimide + hydroxysuccinimide (EDC + NHS), which formed short-lived ether groups, which then reacted with the amino groups of the ligand to form an amide bond. The activation was carried out by passing a mixture of activators from concentrations of 0.05 M EDC/0.2 M NHS through analytical and reference cells for 10 min at a flow rate of 10 μL/min.

Antibodies HTM81 against the Rv0934 antigen were dissolved in 10 mM sodium citrate buffer, pH 4.0, to a concentration of 200 μg/mL. The solution was passed only through the analytical cell; the reference cell remained closed. The passed volume of the solution was 80 μL. Unreacted activated groups in the analytical cell and the reference cell were blocked to prevent nonspecific interaction with 1 M ethanolamine passed for 10 min at a flow rate of 10 μL/min. Antigen samples were passed over the surface of the modified chip through the analytical and control cells at a flow rate of 10 μL/min. At the end of each cycle, the surface was regenerated with a 0.1 M glycine-HCl solution at pH 2.

The equilibrium association constant was calculated in the approximation of equilibrium conditions using the formula:

Req = (Ka·C·Rmax)/(1 + Ka·n·C)

where Ka is the equilibrium binding constant, Req is the level of binding (signal value on the sensogram, Rmax is the maximum possible level of binding, C is the concentration of the added antigen, and n is the steric factor.

The dependence of Req on C was reconstructed in the Scatchard coordinates (1/Req on 1/C). The obtained points were linearized on the Scatchard plot. The point of intersection of the obtained line with the *Y*-axis gives 1/Rmax, and the tangent of the slope of the linearized dependence is 1/(Ka × Rmax). Substituting the obtained 1/Rmax into the expression 1/(Ka × Rmax), the equilibrium association constant was obtained: Ka.

The kinetic dissociation constant was calculated by the formula:

R = R_0_ e ^−kd(t-t^_0_^)^ + Offset

where R is the signal at the time t, R_0_ is the signal at the beginning of the dissociation section (at the time t_0_), and Offset is the background signal. 

The kd was found from the R and R_0_ obtained from the analysis of the dissociation site on the sensograms.

Considering the ratio Ka = k_a_/k_d_; Kd = k_d_/k_a,_ the values of k_a_ and Kd were found.

### 2.9. Determination of the Concentration of Binding Sites on the Gold Nanoparticles Conjugate

The conjugate of gold nanoparticles with the Rv0934 antigen, after synthesis and purification from unbound antigen, was concentrated to an optical density of 5 (at a wavelength of 520 nm) and mixed with a solution containing 80 μg/mL of monoclonal antibodies HTM81. The mixture was incubated for an hour, then the conjugate with the bound antibodies was separated by centrifugation at 8000× *g*. The supernatant containing the antibodies unbound to the conjugate was collected and divided into two parts equal in volume. HTM81 was added to the first solution to a concentration of 10 μg/mL as a calibration. Then, the fluorescence of the solutions was measured upon excitation with light at 280 nm and emission at 350 nm (the maximum fluorescence of tryptophan in the protein). The difference in fluorescence in the first and second solutions corresponds to fluorescence of 10 μg/mL HTM81. The fluorescence value in the second solution divided by the fluorescence value of 10 μg/mL HTM81 and multiplied by 10 gives the concentration of unbound antibodies in the sample. Subtracting this concentration from 80 μg/mL gives the concentration of antibodies bound by the conjugate. This value characterizes the concentration of binding sites on the conjugate.

### 2.10. Numerical Simulation of an Immunochromatographic Assay

Numerical simulation was implemented using COPASI 4.19 software (Biocomplexity Institute of Virginia Tech, University of Heidelberg, and University of Manchester) [47]. 

## 3. Results and Discussion (Theoretical)

### 3.1. Modeling Interactions in an Immunochromatographic System

This paper provides a mathematical description of immunochromatographic serodiagnostics in a format with two antigens using analytical and numerical approaches. In the models, only bivalent immunoglobulins were considered as an analyte, which includes the main class of immunoglobulins: IgG.

At the first stage, a sample containing specific antibodies (Ab) was mixed with a conjugate of an antigen with labeling particles (P) to form complexes of the labeled antigen with one or two valencies of antibodies of PAb and P_2_Ab composition, respectively:

P + Ab = PAb(1)

P + PAb = P_2_Ab (2)

For simplicity, the formation of complexes of other compositions, potentially containing any amount of P and Ab, were not considered. Until the analytical zone with the immobilized antigen was reached, only those reactions were carried out proceed. In this case, because the analytical zone is a thin line through which the reagents flows, it can be assumed that the reactions in the analytical zone do not have time to affect the concentrations of free and labeled immunoglobulins. Therefore, the [P], [Ab], [PAb], and [P_2_Ab] concentrations are determined only by the reactions (1) and (2).

After reaching the analytical zone by the liquid front, three more reactions with the immobilized antigen (Ag) began to proceed in it, expressed by the following Equations:

PAb + Ag = PAbAg(3)

Ab + Ag = AbAg(4)

P + AbAg = PAbAg(5)

The complex of the composition PAbAg was examined. The rate of PAbAg formation determined the kinetic curve of the analysis, and the dependence of the final concentration [PAbAg] on the initial concentration of antibodies [Ab]_0_ provided the calibration curve of the analysis (here and below, the index 0 denotes the initial concentrations of the corresponding components). Each of the reactions, (1)–(5), is characterized by its own kinetic and equilibrium association and dissociation constants. Additionally, two time parameters are introduced to describe the system: t is the analysis time from the moment the sample contacts the labeled antigen, and T is the time from the contact of the sample with the labeled antigen to the contact of the sample with the analytical zone.

**Figure 2:**
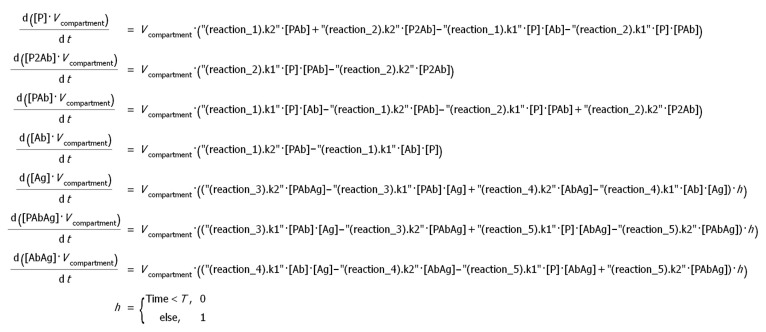
The system of differential equations describing the serodiagnostic lateral flow system with two antigens. k1—kinetic constants of association of the corresponding reaction, k2—kinetic constants of dissociation of the corresponding reaction, *h*—Heaviside function.

To solve the differential equations describing the kinetics of the occurring reactions, a numerical approach was implemented in the COPASI program. The complete system of equations specified in the program is shown in Figure 2:

### 3.2. Formation of PAb and P_2_Ab Complexes

The antibody detection sensitivity in the considered depends on the ratio of [PAb] and [P_2_Ab] concentrations. As the P_2_Ab complex does not have free valences for the formation of a detectable PAbAg complex, the formation of P_2_Ab is an undesirable process. For highly sensitive detection of antibodies in a sample, it is necessary to run the assay under conditions that maintain high [PAb] and low [P_2_Ab] concentrations.

**Figure 3:**
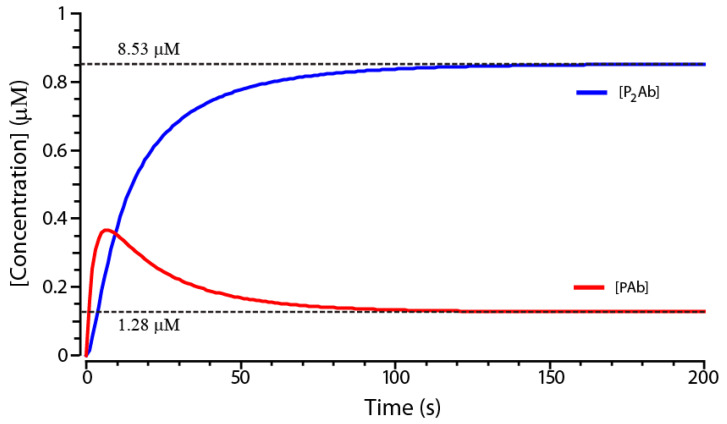
Kinetics of changes in the concentrations of PAb and P_2_Ab complexes (numerical model in the COPASI program). Model parameters: T = 60 s; [P]_0_ = 2×10^−6^ M; [Ab]_0_ = 10^−6^ M; ka1 = ka2 = 10^5^ 1/(M×s); kd1 = kd2 = 2.5×10^−3^ 1/s. Dotted lines indicate equilibrium concentrations of [PAb] and [P_2_Ab].

The system simulated in the COPASI program for arbitrarily chosen concentrations [Ab]_0_ and [P]_0_ gives the kinetic curves of PAb and P_2_Ab formation shown in Figure 3. From the data obtained, it follows that, for the given parameters, the concentration [PAb] will approach the equilibrium value by 90% in 76 s. At the same time, the concentration of [P_2_Ab] will approach the equilibrium by 96%. Assuming that the concentrations of [PAb] and [P_2_Ab] further change insignificantly, the approximation of equilibrium conditions can be applied. This approximation is limited because, with a decrease of the components’ concentrations, it will take more time to reach equilibrium conditions. However, the approximation allows for the deriving of symbolic solutions of equations and determining regularities in the system functioning.

### 3.3. Analytical Model of the Reactions (1) and (2) in Equilibrium Conditions

The equilibrium condition in the system is given by the expressions:

(6) $$\mathrm{Ka}1\;=\;\frac{\left[\mathrm{PAb}\right]}{\left[P\right]\left[\mathrm{Ab}\right]}$$

(7) $$\mathrm{Ka}2\;=\;\frac{\left[P_{2}\mathrm{Ab}\right]}{\left[P\right]\left[\mathrm{PAb}\right]}$$

where Ka1 and Ka2 are equilibrium constants of the reactions (1) and (2), respectively.

Together with the equations of the law of mass conservation, these equations set the equilibrium concentrations of all components of the system:

(8) $${\left[\mathrm{Ab}\right]}_{0}=\;\left[\mathrm{Ab}\right]\;+\;\left[\mathrm{PAb}\right]\;+\;\left[P_{2}\mathrm{Ab}\right]$$

(9) $${\left[P\right]}_{0}=\;\left[P\right]\;+\;\left[\mathrm{PAb}\right]\;+\;2\left[P_{2}\mathrm{Ab}\right]$$

After substituting into Equations (8) and (9) the expressions for PAb and P_2_Ab obtained from (6) and (7), the following was obtained:

(10) $${\left[\mathrm{Ab}\right]}_{0}=\;\left[\mathrm{Ab}\right]\left(1\;+\;\mathrm{Ka}1\left[P\right]\;+\;\mathrm{Ka}1\mathrm{Ka}2{\left[P\right]}^{2}\right)$$

(11) $${\left[P\right]}_{0}=\;\left[P\right]\left(1\;+\;\mathrm{Ka}1\left[\mathrm{Ab}\right]\;+\;2\mathrm{Ka}1\mathrm{Ka}2\left[P\right]\left[\mathrm{Ab}\right]\right)$$

Substituting into equation (11) the expression for [Ab] obtained from (10), an equation with one unknown [P] was obtained:

(12) $${\left[P\right]}_{0}=\;\left[P\right]\left(1\;+\frac{\mathrm{Ka}1\;{\left[\mathrm{Ab}\right]}_{0}\left(1\;+\;2\mathrm{Ka}2\left[P\right]\right)}{1\;+\;\mathrm{Ka}1\left[P\right]\;+\;\mathrm{Ka}1\mathrm{Ka}2{\left[P\right]}^{2}}\right)$$

This equation can be reduced to the form of a cubic equation:

(13) $${\left[P\right]}^{3}\;+\;a{\left[P\right]}^{2}\;+\;b\left[P\right]\;+\;c\;=\;0$$

where a = Kd2 − [P]_0_ + 2[Ab]_0_, b = Kd2×(Kd1 − [P]_0_ + [Ab]_0_) and c = −Kd1×Kd2×[P]_0_.

For convenience, the equilibrium association constants Ka1 and Ka2 were replaced by their reciprocal values—the equilibrium dissociation constants Kd1 and Kd2, respectively.

To solve the cubic equation, Vieta’s trigonometric formulas was used, according to the recommendations of Wang [48].

(14) $$\left[P\right]\;=\;-\frac{a}{3}\;+\;\frac{2}{3}\sqrt{\left(a^{2}\;-\;3b\right)}\cos\frac{\theta }{3}$$

where $\theta \;=\;arc\cos\frac{-2a^{3}\;+\;9ab\;-\;27c}{2\sqrt{{\left(a^{2\;}-\;3b\right)}^{3}}}$.

This solution gives the equilibrium concentration [P]. Substituting it into equation (10), the equilibrium concentration [Ab] was found. Next, substituting the values [P] and [Ab] into equation (6), the equilibrium value [PAb] was found, and the obtained values [PAb] and [P] give the equilibrium concentration [P_2_Ab] from Equation (7).

**Figure 4:**
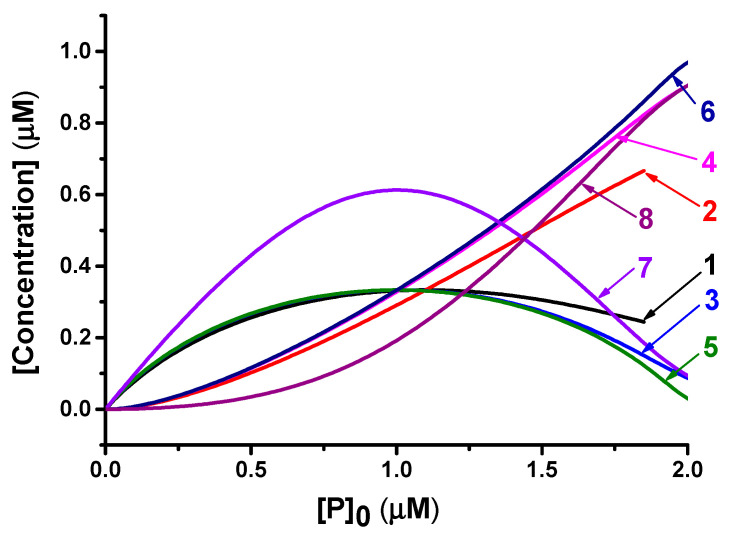
Equilibrium concentrations of PAb and P_2_Ab complexes. Model parameters: [Ab]_0_ = 1 µM; **1.** [PAb] at Kd1 = Kd2 = 0.1 µM; **2.** [P_2_Ab] at Kd1 = Kd2 = 0.1 µM; **3.** [PAb] at Kd1 = Kd2 = 0.01 µM; **4.** [P_2_Ab] at Kd1 = Kd2 = 0.01 µM; **5.** [PAb] at Kd1 = Kd2 = 0.001 µM; **6.** [P_2_Ab] at Kd1 = Kd2 = 0.001 µM; **7.** [PAb] at Kd1 = 0.001 µM, Kd2 = 0.01 µM; **8.** [P_2_Ab] at Kd1 = 0.001 µM, Kd2 = 0.01 µM.

Dependences of equilibrium concentrations [PAb] and [P_2_Ab] on the initial concentration of labeled antigen [P]_0_, shown in Figure 4, demonstrate that, if the [P]_0_ is close to the concentration of the antibodies to be detected, then the equilibrium concentration of [PAb] will be maximum. At lower [P]_0_, the [PAb] concentration decreases due to a lack of labeled antigen, and, at higher [P]_0_, most of the antibodies and labeled antigen were contained in the P_2_Ab complex, which is unable to bind in the assay zone. Moreover, as shown by curves 1, 3, 5, and 7 in Figure 4, this dependence is not contingent on the values and ratio of the equilibrium complexation constants of reactions (1) and (2).

This model predicts the existence of an optimal relationship between [Ab]_0_ and [P]_0_. However, it should be taken into account that the formation of a colored complex in the analytical zone can also occur according to reaction (4), and the maximum concentration of [PAb] promotes only the formation of PAbAg according to reaction (3). Therefore, the optimal ratio of [Ab]_0_ and [P]_0_ in a real system will differ from 1:1. However, in any case, the formation of a P_2_Ab complex decreases the sensitivity of antibody detection. Optimization of system parameters should be aimed at minimizing the effect of reaction (2) on the assay results.

## 4. Results and Discussion (Experimental)

### 4.1. Overview

For the model to adequately describe the real system, it is necessary to substitute into the equations the parameters (complexation constants, component concentrations, time parameters) that are as close as possible to real ones. For this, it is necessary to conduct an experimental study of these parameters.

Time parameters can be measured directly by observing the movement of the liquid front and the colored marker along the test strip membranes. Determination of constants and concentrations requires a more complex technique. To measure them, a model immunochromatographic test system was made for the determination of specific antibodies against the Rv0934 protein. Solutions containing known concentrations of HTM81 monoclonal antibodies against this antigen were used as model samples.

### 4.2. Characterization of the Preparation of Gold Nanoparticles

The dimensional parameters of the obtained gold nanoparticles were evaluated using the transmission electron microscopy. Particle images were obtained at a magnification of 66,000. Table 1 contains the data obtained after analyzing the photographs. The average particle diameter was 22.8 nm for a sample of 210 particles. The resulting preparation was stable, and no aggregates were observed.

### 4.3. Determination of the Constants of Immune Interaction

**Figure 5:**
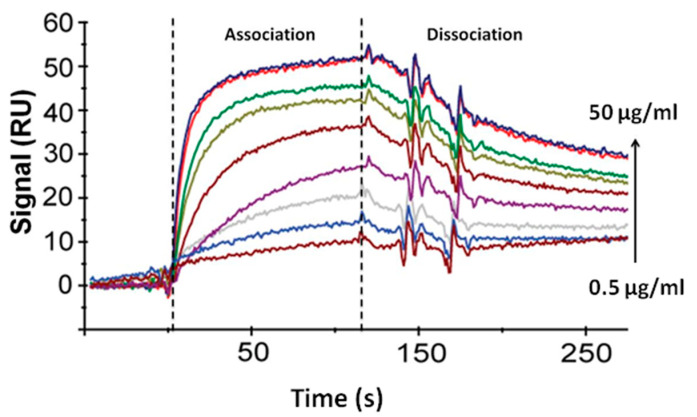
Sensograms of the interaction of the Rv0934 antigen with the surface of the CM5 chip modified with the HTM81 antibodies. Antigen concentration 0.5; 1; 3; 5; 10; 20; 30; 40; and 50 μg/mL. Dotted lines show the beginning and end of the time interval for the flow of antigen solutions through the sensor cell. The signal is the difference between RU values in the analytical cell and the comparison cell.

Sensograms of the interaction of antigen with antibodies were obtained using the surface plasmon resonance sensor BIAcore X, passing over the surface of the optical chip with immobilized antibodies HTM81 solutions containing from 0.5 to 50 μg/mL of Rv0934 antigen (Figure 5).

Using the equilibrium conditions approximation, the equilibrium association constant (Ka) was determined via the Scatchard method based on an analysis of association sensograms. Then, the kinetic dissociation constant (kd) was found from the dissociation curves of immune complexes. After finding Ka and kd, the kinetic association constant (ka) and the equilibrium dissociation constant (Kd) were calculated using the expressions Ka = ka/kd; Kd = kd/ka. Table 2 provides the values of kinetic and equilibrium constants for the studied antigen–antibody pair.

### 4.4. Determination of the Concentration of the Labeled Antigen

When antigen is conjugated to label particles, excessive amounts of antigen are usually used to maximize the binding capacity of the conjugate. In addition, part of the antigen loses binding activity due to the covering of epitopes by label particles. For this reason, the concentration of binding sites on the label conjugate is unknown. To determine this value, a technique based on measuring the fluorescence of tryptophan residues in antibodies was used. Determination of protein concentration based on tryptophan fluorescence was described in detail in our previous work [49].

The Rv0934 protein conjugated to gold nanoparticles (optical density at 520 nm was equal to 2) was mixed with a solution containing 80 μg/mL of antibodies (excessive concentration, since Rv0934 was conjugated to nanoparticles at a concentration of 10 μg/mL per 1 optical density unit). After incubation, the formed complexes with nanoparticles were separated via centrifugation, and the fluorescence of tryptophan in the solution was measured. Comparing the obtained value with the fluorescence of the calibration solution, the concentration of the remaining antibodies was calculated, which was 66 μg/mL. That is, the conjugate bound 14 μg/mL antibodies (93 nM) were at an optical density of 2.

### 4.5. Experimental Verification of Theoretical Relationships

**Figure 6:**
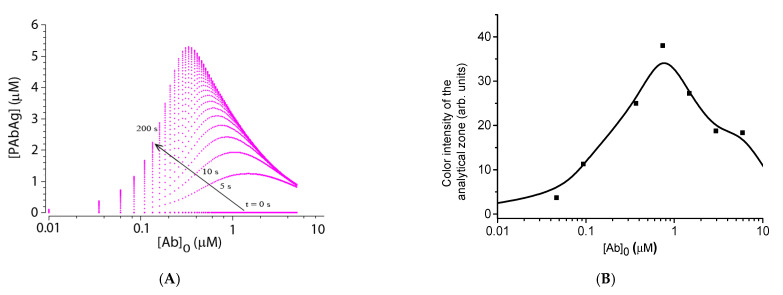
Calibration dependences of analyte determination. (**A**) Theoretical calibration dependence obtained by numerical modeling. Model parameters: T = 60 s; [P]_0_ = 5 × 10^−7^ M; [Ag]_0_ = 10^−5^ M; ka = 10^5^ 1/(M*s); kd = 2.5 × 10^−3^ 1/s. (**B**). Experimental values of the color intensity of the analytical zones after the assay (signal from the Reflekom analyzer).

Theoretical modeling of this system predicts the appearance of the so-called hook effect on the calibration curve—a drop in the signal at high analyte concentrations (Figure 6A). This effect, confirmed experimentally (see Figure 6B), is associated with the fact that the antibodies block all antigen on the marker (gold nanoparticles), and the antibody excess remains in the solution. After the solution reaches the analytical zone, free and label-bound antibodies begin to compete to bind with the immobilized antigen, which leads to a decrease in coloration. Experimental confirmation of the theoretically predicted effect demonstrates the predictive capabilities of the developed model.

As seen in Figure 6, with the indicated parameters of the immunoreactants, the position of the maximum on the calibration curve shifts with time toward lower concentrations, but stabilizes in about 3 min. Note that the model predicts the position of the maximum of the calibration dependence at lower concentrations than observed in the experiment. This may be due to the simplifications introduced into the model, as well as the fact that the concentration of active antibodies in the preparation is somewhat lower than their total concentration.

In real samples, the concentration of specific antibodies against individual antigens rarely exceeds 2 µM, even in hyperimmune sera [36]. Therefore, a decrease in the color intensity to an undetectable limit is unlikely. In addition, the influence of the hook effect on the analysis results can be eliminated by diluting the sample.

**Figure 7:**
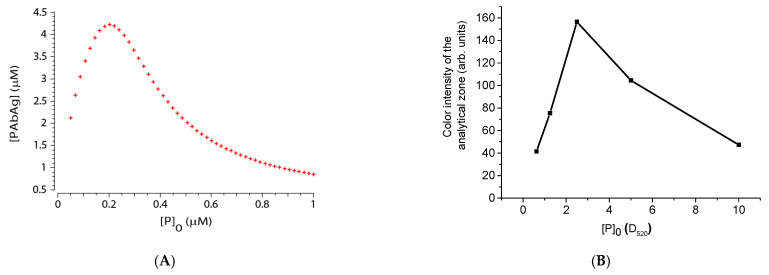
Dependences of analyte determination at different concentrations of labeled antigen (**A**) Theoretical dependence obtained by numerical simulation. Model parameters: T = 60 s; [P]_0_ = 5 × 10^−7^ M; [Ag]_0_ = 10^−5^ M; ka = 10^5^ 1/(M*s); kd = 2.5 × 10^−3^ 1/s. (**B**). Experimental values of the color intensity (signal from the Reflekom analyzer) of analytical zones after the assay at different optical densities (D520) of the label conjugate.

As already noted, one of the ways to control the sensitivity of the assay is to optimize the concentration of the labeled antigen. To verify this assumption, a series of test strips were made with different amounts of added antigen-gold nanoparticle conjugate, and a solution containing 20 μg/mL of antibodies was analyzed. The theoretical dependence calculated in the COPASI program (see Figure 7A) predicts the appearance of a maximum at the point corresponding to a twofold excess of the [P]_0_ concentration relative to the concentration of added antibodies. The experimental dependence had a similar shape and confirmed the existence of the optimal concentration of the labeled antigen (see Figure 7B). However, according to the calculated concentration of the active antigen on nanoparticles, the maximum of the obtained dependence turned out to be lower than that predicted by the model. This fact can also be associated with a partial loss of the antibody activity during storage, as well as with the influence of factors not taken into account by the model—for example, the possibility of the formation of complexes of a more complicated composition.

**Figure 8:**
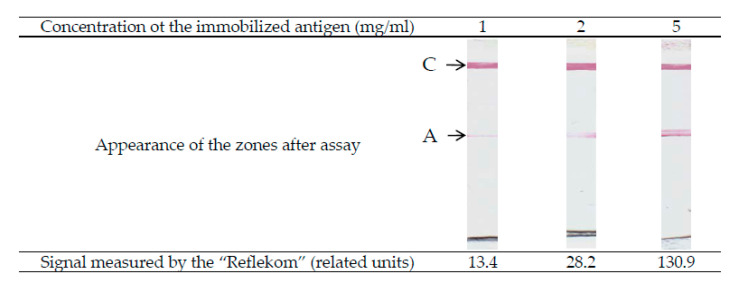
Influence of concentration of the immobilized antigen on the coloration of analytical zone (appearance of working membranes for antibody concentration 200 ng/mL, D520 of the antigen conjugate with gold nanoparticles—2). A—analytical zone; C—control zone.

Another parameter that affects the sensitivity of the system is the concentration of the immobilized antigen in the analytical zone [Ag]_0_. In this case, it is obvious that an increase in [Ag]_0_ leads to an acceleration of the formation of PAbAg both by reactions (3) and (5). Therefore, an increase in the sensitivity and assay signal presupposes the use of the maximum possible antigen concentrations in the analytical zone and is limited only by the sorption capacity of the working membrane (depending on the brand and manufacturer). This assumes, however, the complete absence of nonspecific interaction in the system. If there is such an interaction, it will limit the analytical characteristics of the assay. Figure 8 shows the appearance of the analytical zones of the test strips after the determination of antibodies for three concentrations of the immobilized antigen. According to the study conducted by Merck-Millipore employees, the sorption capacity of immunochromatographic nitrocellulose membranes varies in the range of 4–15 mg/mL (https://www.merckmillipore.com/RU/ru/products/ivd-oem-materials-reagents/lateral-flow-membranes/n6mb.qB.L0YAAAE_gut3.Lxi,nav). This study was carried out using the example of immunoglobulins G; however, since the molecular weight and volume are approximately proportional for globular proteins, this amount will be similar for other proteins.

## 5. Conclusions

Utilizing immunochromatographic serodiagnostics with two antigens appears to be effective for detecting specific immunoglobulins against the background of a multiple excess of total immunoglobulins in the sample. Unlike other formats of immunochromatographic serodiagnostics, this format has not received a theoretical description. In our work, a mathematical model of immunochemical interactions in the system was proposed, which makes it possible to assess the influence of the parameters of immunochemical reactions on the analysis results. This model allows for the formulation of predictions in the behavior of the analytical system and provides recommendations for increasing the sensitivity of antibody detection. The model predicts that the detection limit for antibodies can be reduced if the following are done:

1. Use the concentration of the labeled antigen with approximately twice the concentration of active antibodies in the sample. As the concentration of antibodies against the antigen used is initially unknown, it is necessary to experimentally titrate the amount of labeled antigen to achieve the target sensitivity.
2. Use the highest possible concentration of the immobilized antigen in the analytical zone, but not exceeding the sorption capacity of the working membrane.

The stated regularities were demonstrated in the system for the determination of specific immunoglobulins against the *Mycobacterium tuberculosis* Rv0934 protein. However, the recommendations are of a general nature and can be applied to improve other analytical systems based on the principle of double-antigen sandwich immunochromatographic serodiagnostics.

## Figures and Tables

**Table 1:** Characterization of the particle size and homogeneity of the obtained preparation of gold nanoparticles.

	Major Axis Length (nm)	Minor Axis Length (nm)	Axis Ratio
Average diameter (nm)	22.8	18.5	0.845
Standard deviation (nm)	4.1	2.9	0.095

**Table 2:** Kinetic and equilibrium constants of the interaction of the Rv0934 antigen with monoclonal antibodies HTM81.

ka (1/Ms)	kd (1/s)	Ka (1/M)	Kd (M)
1.08 × 10^5^	2.54 × 10^−3^	4.27 × 10^7^	2.34 × 10^−8^

## Data Availability

Data is contained within the article. The created COPASI file for modelling, data of calculations and verifications are available on request from the corresponding author.
